# UniStArt: A 12-Month Prospective Observational Study of Body Weight, Dietary Intake, and Physical Activity Levels in Australian First-Year University Students

**DOI:** 10.3390/biomedicines10092241

**Published:** 2022-09-09

**Authors:** Nina A. Wilson, Anthony Villani, Sze-Yen Tan, Evangeline Mantzioris

**Affiliations:** 1 Clinical and Health Sciences, University of South Australia, Adelaide, SA 5000, Australia; 2 School of Health and Behavioural Sciences, University of the Sunshine Coast, Sippy Downs, QLD 4556, Australia; 3 Institute for Physical Activity and Nutrition (IPAN), School of Exercise and Nutrition Sciences, Deakin University, Geelong, VIC 3220, Australia; 4 Alliance for Research in Nutrition, Exercise and Activity (ARENA), University of South Australia, Adelaide, SA 5000, Australia

**Keywords:** freshman, weight gain, body composition, diet, physical activity

## Abstract

Background: Students in the United States gain weight significantly during their first year of university, however limited data are available for Australian students. Methods: This 12-month observational study was conducted to monitor monthly body weight and composition, as well as quarterly eating behaviours, dietary intake, physical activity, sedentary behaviours, and basal metabolic rate changes amongst first-year Australian university students. Participants were first-year university students over 18 years. Results: Twenty-two first-year university students (5 males and 17 females) completed the study. Female students gained weight significantly at two, three, and four-months (+0.9 kg; +1.5 kg; +1.1 kg, *p* < 0.05). Female waist circumference (2.5 cm increase at three-months, *p* = 0.012), and body fat also increased (+0.9%, *p* = 0.026 at three-months). Intakes of sugar, saturated fat (both >10% of total energy), and sodium exceeded recommended levels (>2000 mg) at 12-months. Greater sedentary behaviours were observed amongst male students throughout the study (*p* <0.05). Conclusions: Female students are at risk of unfavourable changes in body composition during the first year of university, while males are at risk of increased sedentary behaviours. High intakes of saturated fat, sugars, and sodium warrant future interventions in such a vulnerable group.

## 1. Introduction

Overweight and obesity is a prevalent health problem in Australia across all age groups and 67% of Australians over the age of 18 are considered overweight or obese [1]. Obesity-related comorbidities include cardiovascular diseases, various cancers, musculoskeletal disorders, and type two diabetes [2]. The economic impact of the health consequences of overweight and obesity was reported to be 11.8 AUD billion in 2018 [3], and this is expected to rise significantly in the coming decades.

Research into overweight and obesity in young adult populations is scant, despite 38.4% of young Australians aged 18–24 years being overweight or obese [4]. This high prevalence may be due to the fact that early adulthood is a time in which there are considerable changes in habitual dietary behaviours and reductions in physical activity [5,6,7,8,9,10,11,12]. Preventing the early onset of excessive weight gain is pertinent for the prevention of excessive adiposity and associated comorbidities later in life [13]; therefore, a better understanding of the aetiology of obesity during this time is required to implement early intervention strategies in this vulnerable group.

Changes in living arrangements and decision making, related to lifestyle, often start around early adulthood, a time in which, for many people, also coincides with the commencement of university [14,15]. In Australia, 61% of newly enrolled domestic university students fall within the age range of 15–24 years [16], an age which overlaps with transitioning to independence in many aspects of life, including lifestyle decisions that may impact health status. As such, understanding the lifestyle and weight changes of the Australian young adult population engaged in tertiary education is important and an area that is currently not well-documented in the literature.

In the United States (US), there is a common belief referred to as the ‘Freshman 15’, where college freshmen (the equivalent of first-year university students in Australia) would gain 6.8 kg (15 lbs) during their first year of college [17]. There have been a number of studies designed to investigate this phenomenon in the US. The body of evidence does demonstrate weight gain amongst first-year college students; however, this reported weight gain ranges between 0.7 to 4.2 kg, rather than the alleged 6.8 kg [18,19,20,21,22]. Although this gain is significantly less than the ‘Freshman 15’ claim, first-year university students are susceptible to weight gain; however, this is yet to be investigated in an Australian population. Findings from the US and other countries are difficult to extrapolate to Australian settings, due to the differences in living and environmental arrangements, which could be considered to have unique impacts on dietary intake and physical activity. Furthermore, previous studies from the US have not assessed all factors related to body weight regulation via energy intake and expenditure simultaneously, and it is unclear how habitual dietary intake, physical activity, and other external factors interact and influence the body weight of a university cohort.

For the first time in an Australian setting, this 12-month observational study aimed to monitor changes in body weight among first-year students enrolled in an Australian university and investigate whether dietary intake and physical activity behaviours, in addition to eating behaviour and basal metabolic rate (BMR), impact weight change in these students.

## 2. Materials and Methods

The 12-month prospective observational study followed first-year university students during the academic year from March 2015 to February 2016. Ethics approval for the study was obtained in October 2014 from the Human Research Ethics Committee at the University of South Australia (approval number 0000033624), and the study was registered with the Australia New Zealand Clinical Trials Registry (ACTRN12615001116516).

Participants were recruited over a three-week period: during orientation week (final week of February 2015) through the second week of semester (March 2015). Participants were first-year university students, older than 18 years of age, and had not studied at tertiary level in the previous five years. Additionally, they had no significant medical conditions that could affect key outcome variables of interest, i.e., gastrointestinal disorders, eating disorders, renal disease, and cancer.

Participants were recruited via study flyers placed around two university campuses. Flyers were also provided to university student services for all new students collecting orientation packs. Students were also approached during orientation week, and study information emails were distributed via teaching staff.

Participants who met all inclusion criteria were enrolled into the study. During their first visit, participants were provided with an information sheet, and written informed consent was obtained from all participants prior to study commencement.

The study consisted of one baseline visit and twelve monthly follow-up visits. The baseline visit involved all measurements, including anthropometric measurements, a three-day diet diary (to be completed before the following visit), a series of demographic, physical activity, and eating behaviour questionnaires, and an assessment of basal metabolic rate. The 12-month visit was identical to the baseline visit.

At three, six, and nine months, all anthropometric measurements were assessed, demographic, physical activity, and eating behaviour questionnaires were completed, and participants completed a three-day food diary. During routine, monthly follow-up visits, body weight, and body fat percentage were assessed.

Participants’ demographics, university enrolment (program, course contact hours, and study type), living arrangements, including cooking abilities (scored on a 1–100 scale, with 1 being not confident and 100 being very confident) and money spent on groceries, eating out, and foods bought on campus were obtained through a questionnaire.

Height, weight, waist, and hip circumference measurements were conducted by trained personnel, according to International Standards for Anthropometry and Kinesiology (ISAK) guidelines [23]. Body weight was recorded to the nearest 0.1 kg using calibrated digital scales (Tanita BF-679W Scale and Body Fat Monitor, Tanita Inc, Tokyo, Japan), with participants wearing light clothing, without footwear. Height was measured using a portable stadiometer to the nearest 0.1 cm (Leicester Portable Height Measure, HM-250P Stadiometer, Marsden Weighing Machine Group Limited, Rotherham, UK), with the participant’s head positioned in the Frankfort plane. Height and weight were used to calculate body mass index (BMI  =  mass (kg)/height (m^2^)). The Tanita scale is a single-frequency leg-to-leg bioelectrical impedance analysis (SF-BIA) device (Tanita BF 679W Scale and Body Fat Monitor, Tanita Inc, Tokyo, Japan), and in accordance with the manufacturer’s manual, the subjects stood on the metal contacts in bare feet, and body fat % (BF %) was determined. The measurement was taken in duplicate, with the mean value used in the final analysis. Previous literature has supported the application of SF-BIA as a portable method of assessing BF%, with its acceptable relative agreement against dual-energy X-ray absorptiometry (DEXA) [24,25]. Waist measurements were taken at the point of the visual narrowing, and hip measurements were taken at the furthest protruding point of the buttocks [23], using a flexible steel measuring tape (Lufkin Executive Thinline Flexible Steel Tape W606PM, Apex Tool Group, Saginaw, MI, USA). All measures were taken in duplicate, and the mean was calculated. If the difference between the measurements exceeded 0.5 cm, a third measurement was taken, and the mean of the three measurements was calculated.

Habitual dietary intake was assessed every three months, using a three-day food diary. Participants recorded all food and beverage consumption over three days: two days on campus and one day off campus. Detailed instructions on how to complete the diet diary and an example of a recorded diary were provided to participants. The researcher verbally cross-checked individual food diaries for completeness and acquired additional information, regarding the reported foods and beverages, as required. Habitual dietary intake was analysed using the dietary analysis software, FoodWorks (FoodWorks 8 Professional (2015), Xyris Software Australia Pty Ltd, Brisbane, QLD, Australia), and specifically using the AusFoods and AusBrands databases [26], which provided an analysis of participants’ total energy, macronutrient, and micronutrient (sodium) intakes.

Based on the three-day diet diary, a food group analysis was also performed using the Australian Guide to Healthy Eating (AGHE) food groups as a criterion reference [27]. Using the dietary information provided in the diet diaries, each food and beverage item was categorised into food groups based on the type of food and nutrient profile. The weight of the food and/or beverage was converted into a common serving size of that food, as outlined by the AGHE [27]. All data were entered into an Excel spreadsheet, and the mean serve size for each food group was calculated for on campus university days, off campus days, and for the total three days. Average intake of each food group was calculated against AGHE serving sizes for the total cohort at each time-point.

The validated 27-item International Physical Activity Questionnaire (IPAQ) [28] was used to estimate average weekly physical activity. This questionnaire acquired information on the types of vigorous, moderate, and sedentary physical activities undertaken in the last seven days. The questionnaire was divided into five categories: (1) job-related physical activity, (2) transportation physical activity, (3) housework, house maintenance, and caring for family, (4) recreation, sport, and leisure-time physical activity, and (5) time spent sitting. The IPAQ was scored using the associated scoring document provided and reported as total metabolic equivalent of task (MET) minutes/week. This is a measure that identifies the energy expenditure of certain physical activities, and it was used as the scoring value for this questionnaire [29].

BMR was measured using a previously validated indirect calorimeter method [30] that assessed oxygen and carbon dioxide gas exchanges at rest (TrueOne 2400 Metabolic Measurement System, Parvomedics Inc., Salt Lake City, UT, USA). The coefficient of variation of indirect calorimeter measurements used in this study was 10.5%. The measurement was taken after a 10–12 h fast, with no alcohol or strenuous exercise on the day prior to assessment. Upon arrival, participants rested for 15-min in a seated position. After the resting period, a ventilated hood was placed over the upper body of participants, and gas samples were analysed continuously for 30-min. As per standard REE measurement protocol, only the last 20-min of the data from the 30-min measurement period were used for analysis. The first 10-min were considered a habituation period, and these data were discarded. During the measurement period, using the published Weir equation [31], the BMR measurement was calculated based on the volumes of oxygen consumed (VO_2_) and carbon dioxide produced (VCO_2_).

The validated three-factor eating questionnaire (TFEQ) [32] was used to characterise individuals’ eating behaviours: cognitive restraint, disinhibition, and hunger. The score range for each behaviour was 0–21 for cognitive restraint, 0–16 for disinhibition, and 0–14 for hunger.

Statistical analyses were conducted using IBM Statistical Product and Service Solution software (Version 21, IBM, Chicago, IL, USA) [33]. A ‘per protocol’ (PP) analysis was also conducted using completers’ data only. Chi-square goodness of fit tests (χ^2^) were used to compare categorical demographic responses. General linear model (GLM) for repeated measure ANOVA with Bonferroni correction was used to detect time, sex, and time-by-sex effects, and paired samples t-tests were used to identify changes from baseline. All data are presented as mean ± standard deviation (SD), and statistical significance was set at *p* < 0.05.

## 3. Results

**Figure 1:**
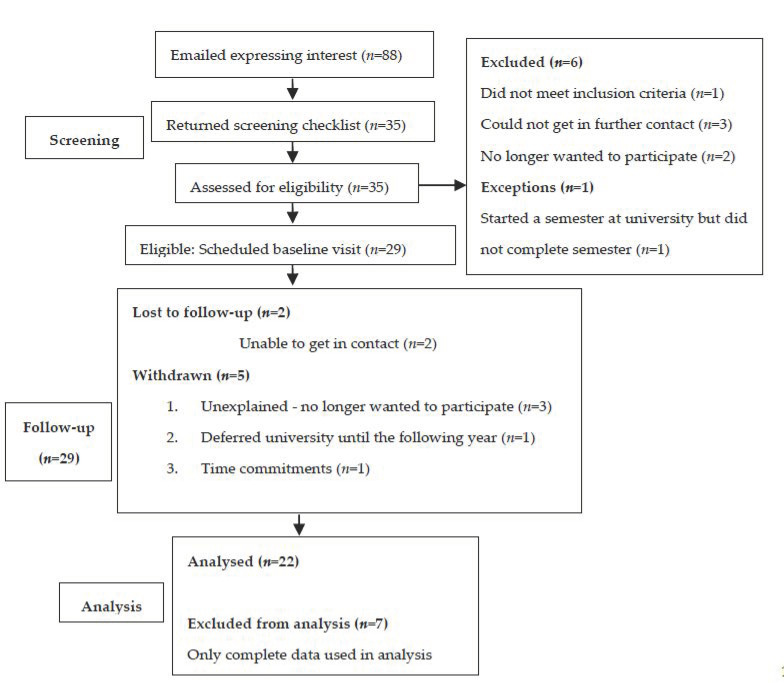
Consort chart outlining the UniStArt study process from participant screening and recruitment until analysis. A total of 29 participants enrolled in the study, while 22 participants were used for analysis.

Eighty-eight participants expressed interest in the study, where 29 participants were eligible and recruited into the study. Of the 29 participants who commenced, 22 participants (*n* = 5 males; *n* = 17 females) completed the study at the 12-month time point (Figure 1).

Baseline characteristics for those who completed the study are presented in Table 1.

**Figure 2:**
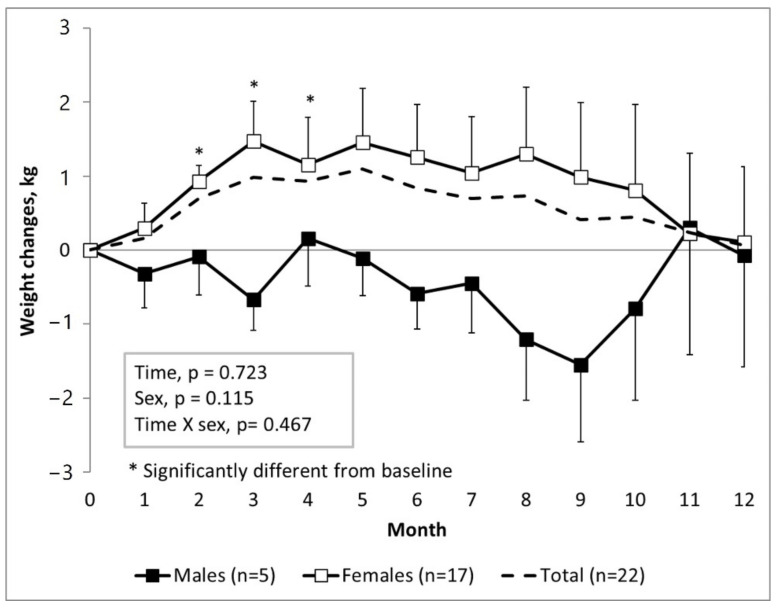
Different trajectories of monthly body weight change between male (*n* = 5) and female (*n* = 17) students during their first year at university. Female students are at risk for weight gain during the first few months, as compared to their male counterparts. Time effects, sex effects, and time x sex effects were analysed using GLM repeated measures ANOVA. * Significantly different from baseline, paired-sample t-tests, *p* < 0.05.

Throughout the first year at university, monthly body weight changed over time in the total sample, but was not statistically significant (Figure 2). However, weight change observed in males, compared to females, followed different trajectories; specifically, significant weight gain from baseline was observed in females from two to four months (paired samples t-test, *p* = 0.004, *p* = 0.006, *p* = 0.037). Towards the end of the study, body weight normalised to baseline weight.

The PP analysis of all study outcomes are presented in Table 2. The use of either statistical analysis methods did not change the findings of our study. Furthermore, since this was an observational study, non-compliance to treatment was not a main concern; therefore, the protocol analysis of 22 study completers was performed and reported in this manuscript.

There was no significant change in body fat % in the total sample; however, there were significant sex effects (*p* = 0.033). Consistent with weight change patterns in females, body fat and waist circumference were significantly higher at three-months than at baseline (paired samples t-test, *p* < 0.05). BMR was higher in males than in females (sex effects, *p* = 0.003) and reduced over 12-months (time effects, *p* = 0.001); this decrease in BMR between sexes approached statistical significance (interaction effects, *p* = 0.062).

Mean daily energy intake significantly decreased from baseline in the total sample at 9- and 12-months (paired samples t-test, *p* < 0.05) and was predominantly attributed to a significant reduction in energy intake in females at these time points (sex effects, *p* = 0.001). Lower total energy intake was also contributed to by a significant reduction in total fat, saturated fat, carbohydrate, and sugar consumption. Conversely, male students did not alter dietary intake significantly, but sodium intake was significantly higher in males than in females (sex effects, *p* = 0.042). Total daily sugar, saturated fat, and sodium intakes exceeded the recommended levels (less than 10% of total energy intake for saturated fat and sugar, as well as a suggested dietary target of 2000 mg of sodium) in all participants.

No significant changes in eating behaviour scores were observed, but hunger was found to be higher in males than females (sex effects, *p* = 0.013). Physical activity remained relatively stable in all participants, but a significant increase in sitting time was observed in male students at 12-months of the study (interaction effects, *p* = 0.037).

The food group analysis (Table 3) indicated that, at 12-months, there was a significant reduction in discretionary sweet food intake (GLM repeated measures ANOVA, *p* = 0.03). Analyses further revealed that students did not eat differently between university and non-university days. Moreover, dietary intake remained unchanged throughout the study, except for a significant reduction in discretionary sweet food intake at 12-months (GLM repeated measures ANOVA, *p* = 0.026).

When compared with the AGHE recommendations, participants consumed less than the recommended daily serves for wholegrain cereals, fruit, vegetables, protein foods, milk, and milk alternatives throughout the 12-months. Consumption of total discretionary foods exceeded recommendations at baseline, 6-months and 12-months (4.9 ± 4.6, 4.3 ± 5.0, and 3.5 ± 4.2 serves/day respectively).

## 4. Discussion

To the best of our knowledge, this is the first Australian study investigating the dynamic interactions between body weight, dietary intake, physical activity levels, and eating behaviours of first-year undergraduate university students for 12-months. We observed that female Australian first-year university students are at risk of unfavourable body composition changes, specifically favouring an increase in central adiposity. Male students’ body weight reduced and returned to baseline values again at the end of the study, possibly due to increased sedentary behaviours. Whether these changes in body composition apply to first-year students only or to all students should be investigated in a larger sample to identify whether different strategies may be required to help male and female students maintain a healthy body weight.

Differential weight change trajectories between male and female students were an interesting and novel finding. Weight gain in females was consistent with findings from previous studies in the US, United Kingdom, and Belgium, in which weight and fat mass both increased in first-year university students over the first few months of the academic year [34,35,36]. However, why this was not observed in male students in our sample warrants further investigations, although the small sample size of males in this study may have influenced our ability to identify significant changes. It is also important to note that all students’ weight returned to baseline weight by 12-months, which highlights the importance of the regular surveillance of body weight in such studies. Several other studies that have identified significant weight gain in this population have only conducted observations over short periods of time (e.g., three-months or less) and, therefore, may not have captured periods later on in the year, in which students’ body weight regulated and returned to baseline [35,37,38,39]. Findings from previous research have also indicated an overall decrease in energy and total fat intake in first-year university students [18,40,41,42], but this was observed only in female students in our study. The time in which body weight normalised in females was around the time of university breaks in the summer. This may indicate that females have more time during breaks to maintain a healthy lifestyle—this is in contrast to previous literature, which suggested that females gain weight over university holiday break periods [43]. Additionally, stress during semester and exam times may have resulted in weight gain amongst females [44,45,46,47].

There was no significant change in male students’ body weight, although weight appeared to decrease and was later regained, resulting in males returning to baseline weight. This regain in body weight may have been attributed to an increase in sitting time at the 12-month study time-point and the associated decrease in BMR observed amongst males. Significant increases in sedentary behaviours have only been observed in two previous studies, and these increases occurred in males also [47,48]. Greater sitting time in males in the present study may be due to the general university lifestyle, as participants became more accustomed to spending time sitting in lectures, tutorials, and studying; however, why this increase was shown in males only is unknown. This finding is concerning, as sedentary behaviours, independent of physical activity, are associated with increased risks of weight gain, cardiovascular diseases, type two diabetes, and overall mortality risk [49,50,51,52]. Therefore, the provision of organised sport and opportunities to participate in physical activity by universities may be beneficial to males within this population [53,54].

The maintenance of body weight in males and females in this cohort may suggest that the impact of the transition from high school to university in an Australian population is not as strong as previously thought. Differences in the university culture and environments between countries, in which many Australian students attend local universities and, hence, remain living at home with parents, may be accountable. This was the case in our study, as the majority of participants lived at home both prior to and during university, and this did not significantly change. Importantly, most of the participants specified that their parents, partner, or housemate undertook the grocery shopping; thus, dietary intake was still largely influenced by family and friends. Previous evidence has highlighted the potential dietary influences when living at home and suggests that those students who move away from home during university have increasingly poorer dietary quality [15,55,56]. Previous evidence has also suggested that those students living on campus gain more weight than those living off campus [57], and peer influence may also be a factor in weight changes during university [58,59].

Although previous studies have reported different eating behaviour patterns among first year university students [60,61], we did not observe this in our study. However, dietary intakes in this study exceeded the current guidelines for saturated fat, sugar, and sodium [62,63,64]. Previous research in the US has similarly indicated a cause for concern in the diet quality of first-year university students [18]; given the high levels of saturated fat, sugar, and sodium consumed in this sample, further analysis of dietary patterns in this vulnerable cohort is warranted. This is particularly important, as prolonged exposure to high intakes of sodium, sugar, and saturated fat may increase the risk of serious health problems, including cardiovascular diseases [65,66,67]. Early identification of these increased intake levels may warrant early intervention in preventing obesity and other health problems later in life.

The food group analysis in the present study also highlighted an inadequate intake of dairy, fruit, and vegetables. These findings are consistent with previous literature in university students from other countries [9,56,68,69], and they are of concern, as low intakes of fruit and vegetables are associated with cardiometabolic disease risk, including cardiovascular disease and type two diabetes.

## 5. Conclusions

This study has a number of strengths. First, it was a prospective observational study allowing for the regular surveillance of variables in three main domains: (1) anthropometric measurements, (2) physical activity, and (3) dietary habits, as well as the eating behaviours and BMR of first-year university students. For this reason, findings allowed the assessment of how these three aspects interacted to regulate body weight. Despite the small sample size, the long observation period was able to provide insight into weight changes over time and identified that weight returned to normal, an important factor that shorter studies may have overlooked. Results from this study will provide useful data for future follow-up studies and serve as a basis for consideration for future interventions in Australian universities, including to inform and aid in the development of behavioural intervention programs targeted at preventing weight gain, improving diet quality, and increasing physical activity in first-year university students.

There were also limitations to this research. Firstly, there was a small sample size of 22 participants. The small sample size was due to the difficulties in recruitment and short recruitment period, which was the result of the time constraints involved in capturing the academic year; future studies should consider this for recruitment purposes. Moreover, the increases in body fat percentage observed in this study may be the result of violation of the principals of the BIA methodology, relating to day-to-day variations in the hydration status and timing of the body composition assessments [70]; nevertheless, a coefficient of variation under repeated measures was performed to minimise this possibility and ensured the reliability of the BIA method in this study (variation of 1.0%). A small sample size may have limited the statistical power to perform further analyses, based on various demographic characteristics. In the future, a larger sample size could improve the significance and relevance of the results. Meanwhile, the majority of the participants were students at one university campus (*n* = 21), a campus in which many of the programs of study are health-related, highlighting the potential issues with the generalisability of the study findings. Thus, it is possible that the participants involved in the study may have been more likely to participate in such a study, as it was in line with their health and future career interests. In turn, this may have influenced their dietary and physical activity habits, and participants may also have monitored their weight more closely than the average university student because of their interests. Future studies should, therefore, increase the sample size, include students from all faculties, and perhaps also include or continue to follow students from second or third years, in order to determine whether weight and lifestyle changes are unique in first-year students only.

This observational study of first-year Australian university students was the first of its kind and demonstrated that female students may be at risk for weight gain during the first few months of the academic year; however, our findings did not support the “Freshman 15” phenomenon from the US. An increase in sedentary behaviours was also observed in males, and all students exceeded the recommended limits for saturated fat, sugar, and sodium intakes, which warrants further investigation into early nutrition intervention in this vulnerable population.

## Figures and Tables

**Table 1:** Baseline demographics and characteristics of 22 first-year university students from an Australian university.

Characteristics (*n* = 22)	Mean ± SD
**Clinical characteristics**	
Sex (M/F)	5 males & 17 females
Age (years)	21.1 ± 6.8
Height (cm)	167.6 ± 9.3
Weight (kg)	65.0 ± 19.2
Body fat (%)	24.0 ± 9.2
Body mass index (kg/m^2^)	22.9 ± 5.4
Waist circumference (cm)	75.2 ± 13.2
Hip circumference (cm)	97.9 ± 11.9
Basal metabolic rate (kcal/day)	1392 ± 376
**Demographic characteristics**	*n* **(%)**
*Marital status*	
Single	20 (90.9%)
Married	1 (4.5%)
Divorced	0 (0%)
De facto	1 (4.5%)
*Smoking status*	
Current smoker	1 (4.5%)
Non-smoker	20 (90.9%)
Smoked previously	1 (4.5%)
*Employment status*	
Employed	8 (36.4%)
*Living situation prior to university*	
Alone	1 (4.5%)
With parents	18 (81.8%)
With partner	2 (9.1%)
With friends (rent a room)	1 (4.5%)
Residential college	0 (0%)
*Current living situation*	
Alone	2 (9.1%)
With parents	13 (59.1%)
With partner	4 (18.2%)
With friends (rent a room)	2 (9.1%)
Residential college	1 (4.5%)
Pre-university vs. current living situation ^1^, *p* = 0.515	
*Enrolment type*	
Full-time (*n* = 20)	90.9
Part-time (*n* = 2)	9.1
*Grocery shopping*	
Myself (*n* = 5)	22.7
Parents/partner/housemate (*n* = 16)	72.7
Homestay/residential college (*n* = 1)	4.5

^1^ Chi-square test. *p* value suggests that living arrangement prior to vs. after university commencement remained unchanged.

**Table 2:** Anthropometric, physical activity, dietary intake, and eating behaviour in 22 students during their first year at an Australian university.

	Baseline	3-Months	6-Months	9-Months	12-Months	Time,	Sex,	Time X sex, *p*
	Mean ± SD	Mean ± SD	Mean ± SD	Mean ± SD	Mean ± SD	*p*	*p*	
**Anthropometry**								
Weight, kg	65.0 ± 19.2	66.0 ± 19.8 ^a^	65.8 ± 19.7	65.4 ± 19.9	65.1 ± 20.1	0.723	0.115	0.467
M (*n* = 5)	78.3 ± 30.1	77.6 ± 30.8	77.7 ± 29.5	76.7 ± 28.0	78.2 ± 28.4			
F (*n* = 17)	61.1 ± 13.6	62.6 ± 15.0 ^a^	62.4 ± 15.3	62.1 ± 16.5	61.2 ± 16.1			
Body fat, %	24.0 ± 9.2	25.3 ± 10.4	24.3 ± 9.6	24.0 ± 9.2	24.2 ± 9.2	0.199	0.033 *	0.381
M (*n* = 5)	16.9 ± 9.1	19.3 ± 15.5	16.4 ± 9.1	16.2 ± 9.1 ^a^	17.5 ± 8.7			
F (*n* = 17)	26.1 ± 8.4	27.0 ± 8.3 ^a^	26.6 ± 8.7	26.2 ± 8.1	26.2 ± 8.6			
Waist, cm	75.2 ± 13.2	76.8 ± 12.2	76.4 ± 12.8	75.9 ± 12.9	76.0 ± 13.2	0.855	0.167	0.235
M (*n* = 5)	83.9 ± 17.8	82.2 ± 19.0	82.7 ± 18.4	82.3 ± 17.5	83.9 ± 17.1			
F (*n* = 17)	72.7 ± 10.9	75.2 ± 9.6 ^a^	74.6 ± 10.7	74.0 ± 11.1	73.7 ± 11.4			
Hip, cm	97.9 ± 11.9	99.1 ± 12.2	99.3 ± 12.8	98.2 ± 13.3	97.3 ± 13.3	0.591	0.698	0.31
M (*n* = 5)	100.7 ± 12.8	12.2 ± 12.6	100.1 ± 13.2	100.0 ± 11.7	100.5 ± 11.4			
F (*n* = 17)	97.1 ± 11.9	98.7 ± 12.5	99.1 ± 13.1	97.6 ± 14.0	96.3 ± 14.0			
**Dietary intake**								
Energy intake, kJ	8915 ± 2437	9245 ± 2978	8532 ± 2862	7679 ± 2393 ^a^	7606 ±2060 ^a^	0.073	0.001 ***	0.83
M (*n* = 5)	10339 ± 3687	11969 ± 2575	10825 ± 1822	9305 ± 3513	9584 ± 2465			
F (*n* = 17)	8496 ± 1892	8444 ± 2643	7857 ± 2791	7201 ± 1834 ^a^	7024 ± 1572 ^a^			
Protein intake, g	85.3 ± 31.5	94.9 ± 32.7	90.3 ± 32.5	79.0 ± 29.9	83.0 ± 30.7	0.095	0.126	0.568
M (*n* = 5)	91.5 ± 34.7	115.9 ± 31.1 ^a^	113.9 ± 18.2	85.2 ± 35.0	96.3 ± 31.9			
F (*n* = 17)	83.5 ± 31.3	88.7 ± 31.3	83.5 ± 32.9	77.1 ± 29.2	79.1 ± 30.1			
Fat intake, g	85.3 ± 29.2	82.3 ± 27.7	74.4 ± 25.8	65.9 ± 23.0 ^a^	64.5 ± 20.1 ^a^	0.031 *	0.184	0.99
M (*n* = 5)	96.6 ± 46.1	89.1 ± 33.2	84.0 ± 13.1	71.0 ± 32.7	71.1 ± 16.2			
F (*n* = 17)	81.9 ± 23.1	80.3 ± 26.8	71.6 ± 28.2	64.4 ± 20.5 ^a^	62.5 ± 21.1 ^a^			
Saturated fat intake, g	30.7 ± 10.4	30.7 ± 11.9	26.9 ± 12.6	23.3 ± 8.5 ^a^	23.1 ± 9.4 ^a^	0.083	0.128	0.983
M (*n* = 5)	34.3 ± 13.1	33.9 ± 13.4	31.4 ± 11.2	25.1 ± 10.5	28.0 ± 5.3			
F (*n* = 17)	29.7 ± 9.6	29.7 ± 11.6	25.6 ± 12.9	22.8 ± 8.2 ^a^	21.7 ± 10.0 ^a^			
CHO intake, g	242.2 ± 77.4	260.6 ± 111.4	240.0 ± 91.6	218.5 ± 81.8	211.5 ± 80.9	0.122	<0.001 ***	0.445
M (*n* = 5)	290.8 ± 103.0	385.6 ± 108.1	329.4 ± 80.7	292.5 ± 109.3	299.1 ± 120.8			
F (*n* = 17)	227.9 ± 65.3	223.8 ± 84.0	213.8 ± 78.4	196.7 ± 59.8	185.8 ± 42.9 ^a^			
Sugar intake, g	96.1 ± 34.0	90.1 ± 35.9	87.9 ± 30.6	84.4 ± 35.2	71.0 ± 27.7 ^a^	0.028 *	0.081	0.397
M (*n* = 5)	113.8 ± 51.1	122.1 ± 18.9	92.5 ± 31.4	98.6 ± 43.4	78.0 ± 32.2			
F (*n* = 17)	90.9 ± 27.1	80.7 ± 34.5	86.6 ± 31.2	80.2 ± 32.7	69.0 ± 26.9 ^a^			
Sodium, mg	2440 ± 767	3029 ± 1558	2827 ± 1449	2673 ± 2140	2542 ± 953	0.115	−0.042 *	0.241
M (*n* = 5)	2775 ± 959	4717 ± 1344 ^a^	3673 ± 1134	3063 ± 1994	3219 ± 406			
F (*n* = 17)	2341 ± 704	2532 ± 1258	2579 ± 1465	2558 ± 2226	2342 ± 983			
**Physical activity**								
PA, MET mins	3704 ± 3260	2896 ± 2301	4824 ± 3479	5897 ± 9910	3770 ± 1840	0.265	0.258	0.645
M (*n* = 5)	5985 ± 2197	3688 ± 1782	7481 ± 3531	7282 ± 8787	3989 ± 1270			
F (*n* = 17)	3033 ± 3263	2662 ± 2430	4042 ± 3149	5490 ± 10432	3705 ± 2005			
Sitting time, min/wk	2360 ± 912	2527 ± 1095	2729 ± 1007	2340 ± 1066	2663 ± 1070	0.09	0.827	0.037 *
M (*n* = 5)	1944 ± 757	2172 ± 1170	2712 ± 1187	2676 ± 1383	3444 ± 1209 ^a^			
F (*n* = 17)	2483 ± 937	2631 ± 1086	2734 ± 989	2241 ± 983	2434 ± 944			
**Basal metabolic rate**								
BMR, kcal/d	1392 ± 376	Not measured	Not measured	Not measured	1293 ± 338	0.001 ***	0.003 **	0.062
M (*n* = 5)	1817 ± 458				1613 ± 503			
F (*n* = 16)	1259 ± 230				1193 ± 201			
**Eating behaviours**								
Restraint	8.2 ± 5.1	8.0 ± 5.7	7.2 ± 5.4	7.7 ± 5.3	7.4 ± 5.0	0.347	0.159	0.722
M (*n* = 5)	6.2 ± 3.6	5.0 ± 2.1	4.6 ± 2.7	4.2 ± 1.6	4.4 ± 1.5			
F (*n* = 16)	8.8 ± 5.4	8.9 ± 6.1	7.9 ± 5.9	8.7 ± 5.6	8.3 ± 5.4			
Hunger	5.5 ± 3.1	5.8 ± 3.5	6.0 ± 3.6	5.8 ± 3.1	5.5 ± 3.6	0.671	0.013 *	0.548
M (*n* = 5)	8.0 ± 2.9	8.8 ± 3.6	9.4 ± 3.8	8.2 ± 3.1	9.0 ± 4.3			
F (*n* = 16)	4.8 ± 2.8	4.9 ± 3.0	5.0 ± 3.0	5.1 ± 2.9	4.5 ± 2.8			
Disinhibition	5.6 ± 2.4	6.0 ± 2.6	6.0 ± 3.1	5.4 ± 2.6	5.3 ± 2.8	0.264	0.879	0.19
M (*n* = 5)	5.6 ± 2.4	5.2 ± 3.3	7.0 ± 4.4	4.6 ± 3.4	5.2 ± 3.8			
F (*n* = 16)	5.6 ± 2.5	6.3 ± 2.5	5.6 ± 2.6	5.7 ± 3.4	5.3 ± 2.6			

PA—physical activity; CHO—carbohydrate; BMR—basal metabolic rate. **^a^** Significantly different from baseline values, paired-samples t-test, *p* < 0.05. * Significant effects, general linear model for repeated measures ANOVA, *p* < 0.05. ** Significant effects, general linear model for repeated measures ANOVA, *p* < 0.01. *** Significant effects, general linear model for repeated measures ANOVA, *p* < 0.001.

**Table 3:** Australian Guide to Healthy Eating (AGHE) food group analyses from the diet diaries of *n* = 22 university students during their first year at an Australian university.

Food Group	AGHE Serving Size	Baseline		6-Months		12-Months		Time *p*	Interaction *p*
		Mean	SD	Mean	SD	Mean	SD		
**Bread and cereals**	30–125 g	3.9	2.4	3.5	1.8	3.5	1.8	0.844	0.268
**Fruit**	150 g	0.9	1.1	0.8	0.7	0.5	0.6	0.205	0.428
**Processed fruit**	30 g/125 mL	0.5	0.8	0.4	0.6	0.2	0.5	0.351	0.173
**Fresh/frozen veg**	75 g	2.8	2.3	3	2.2	2.6	2	0.864	0.571
**Canned veg**	75 g	0.2	0.4	0.2	0.4	0.3	0.5	0.847	0.468
**Legumes**	150 g	0.1	0.2	0.1	0.2	0	0.1	0.407	0.07
**Milk and alternatives**	40–250 g/mL	1.1	0.9	1	0.7	1	0.8	0.894	0.734
**Low-fat milk**	40–250 g/mL	0.3	0.6	0.2	0.3	0.2	0.3	0.129	0.743
**Meat and alternatives**	65–80 g	0.4	0.7	0.6	0.7	0.6	0.4	0.49	0.609
**Fatty meat**	60 g (processed)-65 g	0.2	0.3	0.3	0.6	0.1	0.2	0.389	0.464
**Lean meat/poultry**	65 g lean/80 g poultry	1	1.2	0.9	0.8	1.1	1.6	0.978	0.152
**Fish and seafood**	100 g	0.3	0.4	0.4	0.5	0.2	0.3	0.108	0.134
**Eggs**	2 large eggs (120 g)	0.2	0.3	0.1	0.2	0.1	0.2	0.14	0.566
**Nuts and seeds**	30 g	0.2	0.4	0.2	0.4	0.2	0.4	0.792	0.596
**Unsaturated oils**	5 g	2.5	3.2	1.8	1.9	1.7	1.8	0.293	0.255
**Alcohol**	100 mL wine 285 mL full strength beer 60 mL port or sherry 30 mL spirits	0.2	0.6	0.1	0.3	0.2	0.4	0.309	0.079
**Discretionary sweet**	600 kJ	2.1	1.4	1.7	1.9	1	1	0.026 *	0.972
**Discretional savoury**	600 kJ	2.4	2.1	2.2	2.3	1.9	2.2	0.587	0.582
**Soft drink**	375 mL	0.2	0.5	0.3	0.5	0.4	0.6	0.345	0.947
**Water**	2600 mL	1360	627	1101	811	1228	903	0.069	0.96
**Tea/Coffee**	Not stated	99	210	71	280	109	249	0.298	0.903

* Significant effects, general linear model for repeated measures ANOVA, *p* < 0.05.

## Data Availability

Access to all necessary data and materials are provided within the manuscript.
